# Evaluation of the new AJCC staging system for resectable hepatocellular carcinoma

**DOI:** 10.1186/1477-7819-9-114

**Published:** 2011-09-30

**Authors:** Chih H Cheng, Chen F Lee, Tsung H Wu, Kun M Chan, Hong S Chou, Ting J Wu, Ming C Yu, Tse C Chen, Wei C Lee, Miin F Chen

**Affiliations:** 1 Department of Surgery, Chang Gung Memorial Hospital, Linkou, Chang Gung University Medical School, Taoyuan, Taiwan; 2 Graduate Institute of Clinical Medical Sciences, Chang Gung University, Taoyuan, Taiwan; 3 Department of Pathology, Chang Gung Memorial Hospital, Chang Gung University Medical School, Taoyuan, Taiwan

**Keywords:** American Joint Committee on Cancer, Tumor encapsulation, Hepatocellular carcinoma, Partial hepatectomy, TNM-7

## Abstract

**Background:**

The aim of this study was to assess the validity of the 7^th ^edition of the American Joint Committee on Cancer (AJCC) TNM system (TNM-7) for patients undergoing hepatectomy for hepatocellular carcinoma (HCC).

**Methods:**

Partial hepatectomies performed for 879 patients from 1993 to 2005 were retrospectively reviewed. Clinicopathological factors, surgical outcome, overall survival (OS), and disease-free survival (DFS) were analyzed to evaluate the predictive value of the TNM-7 staging system.

**Results:**

According to the TNM-7 system, differences in five-year survival between stages I, II, and III were statistically significant. Subgroup analysis of stage III patients revealed that the difference between stages II and IIIA was not significant (OS, *p *= 0.246; DFS, *p *= 0.105). Further stratification of stages IIIA, IIIB and IIIC also did not reveal significant differences. Cox proportional hazard models of stage III analyses identified additional clinicopathological factors affecting patient survival: lack of tumor encapsulation, aspartate aminotransferase (AST) values > 68 U/L, and blood loss > 500 mL affected DFS whereas lack of tumor encapsulation, AST values > 68 U/L, blood loss > 500 mL, and serum α-fetoprotein (AFP) values > 200 ng/mL were independent factors impairing OS. Stage III factors including tumor thrombus, satellite lesions, and tumor rupture did not appear to influence survival in the stage III subgroup.

**Conclusions:**

In terms of 5-year survival rates, the TNM-7 system is capable of stratifying post-hepatectomy HCC patients into stages I, II, and III but is unable to stratify stage III patients into stages IIIA, IIIB and IIIC. Lack of tumor encapsulation, AST values > 68 U/L, blood loss > 500 mL, and AFP values > 200 ng/mL are independent prognostic factors affecting long-term survival.

## Background

Hepatocellular carcinoma (HCC) is one of the most common cancers observed world-wide [1,2]. This form of cancer is especially prevalent in Taiwan due to the high number of carriers of chronic hepatitis B and is commonly observed among subjects in the 6^th ^decade [3,4].

Several therapeutic approaches have been developed for the treatment of HCC. Surgical resection is the treatment of choice for resectable forms of the disease. In addition to liver transplantation, resection is advocated as a potentially curative treatment. With recent improvements in surgical techniques and postoperative management, hospital mortalities have been reduced to values approaching zero, with morbidities ranging from 10 to 25% [5-7]. However, long term prognoses vary widely due to the lack of coherent staging systems.

Several staging systems with different prognostic predictors and treatment algorithms have been proposed. The most commonly used are the Barcelona Clinic Liver Cancer [BCLC] [8], Cancer of the Liver Italian Program [CLIP] [9], and Tumor-Node-Metastasis [TNM] [10] systems in Europe and in the United States, the Okuda [11] and Japan Integrated Staging [JIS] [12] scores in Japan, and the Chinese University Prognostic Index [CUPI] [13] staging system in China. However, unlike other types of cancer, the prognosis of HCC is determined not only by the anatomical involvement and growth pattern of the tumor but also by pathophysiological features such as the presence of liver cirrhosis and the grade of residual liver function [14-17].

**Figure 1:**
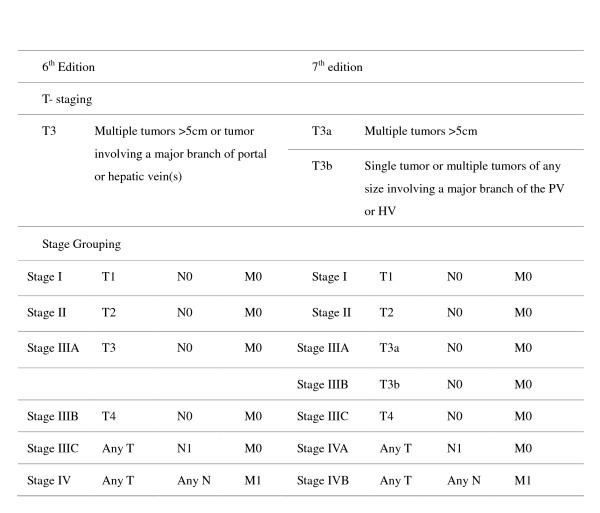
**The 6^th ^and 7^th ^editions of the American Joint Committee on Cancer (AJCC) TNM staging system**.

The American Joint Committee on Cancer (AJCC)/International Union Against Cancer (UICC) TNM system is one of the most commonly used staging systems. TNM staging for HCC is focused on the impact of extrahepatic spread, lymph node involvement, and tumor characteristics such as size (5 cm), vascular invasion, and satellite lesions. The new 7^th ^edition (TNM-7) of the AJCC/UICC TNM system [10], which was introduced in 2009, is a modified version of the 6^th ^edition (TNM-6) of this system. The major modifications of this new system are: stage IIIA includes only multiple tumors or any tumor larger than 5 centimeters (T3a); stage IIIB includes only tumors of any size involving a major portal vein or hepatic vein (T3b); and T4 status is shifted to stage IIIC (Figure 1). These modifications bring new issues to ongoing debates over tumor staging. The purpose of the present study, therefore, was to assess the validity of the TNM-7 staging system for a large series of patients with resectable HCC at a single center.

## Materials and methods

### Patients

Between January 1993 and June 2005, 879 patients with HCC underwent hepatic resections at the Linkou Chang Gung Memorial Hospital. All enrolled patients were staged according to the 7th edition of the AJCC/UICC TNM system and analyzed retrospectively. Because this study was aimed to evaluate the prognostic value of this new TNM system for resectable HCC, patients classified with stages IVA and IVB were excluded. Clinicopathological factors for these patients were also analyzed. Patients with incomplete clinical data or who were lost follow-up were excluded.

### Preoperative assessment

Before 1995, the preoperative evaluation relied on preoperative liver function and Child-Pugh status of the patients. After 1995, the algorithm for selecting patients for hepatectomy was according to Makuuchi's criteria and indocyanine green retention rate at 15 minutes (ICG R15) [18,19].

### Operative technique

During surgery, the abdomen was explored through a subcostal incision with a midline xyphoid extension or through a Mercedes star incision. Intraoperative ultrasonography was routinely performed in order to confirm resectability and evaluate the relationship between the resection line and major vascular structures. Inflow control with the Pringle maneuver was commonly applied intermittently. Hemivascular control was performed in selected right or left hepatectomies. Before 2002, all the resections were performed with peon-crushing technique. After that period, the liver parenchyma was divided with clamp-crushing technique or ultrasonic dissector (CUSA) according to the surgeon's preference, without influencing the postoperative outcome as previously reported [20,21].

### Follow up

After surgery, all patients were followed every 3 months in the out-patient clinic with regular determinations of serum α-fetoprotein (AFP) concentration and with imaging studies, such as abdominal ultrasonography or computed tomography (CT). When recurrence was suspected, abdominal CT or hepatic angiography was performed. Disease free survival (DFS) was defined as the period from the date of hepatectomy to the date of recurrence as detected by imaging studies. Overall survival (OS) was defined as the period from the date of hepatectomy to the date of death.

### Statistical analyses

Survival rates were calculated using the Kaplan-Meier method, and survival curves were compared using the log-rank test. Continuous data were expressed as medians with interquartile ranges. To identify the clinicopathological factors with independent prognostic significance, multivariate analysis was performed using a Cox regression model. In all analyses, a *p *value of less than 0.05 was considered statistically significant. All statistical analyses were performed using SPSS version 13.0 software (SPSS Inc., Chicago, IL, USA).

## Results

### Long-term outcome of resectable HCC as stratified by TNM-7 staging

The clinicopathological characteristics of 879 patients with resectable HCC are summarized in Table 1. The operative mortality rate was 4.0% (*n *= 35) and the surgical complication rate was 26.5%. Major hepatectomy, defined as the resection of more than three segments, was performed in 375 (42.6%) patients and minor hepatectomy was performed in 504 (57.3%) patients. Among these patients, 844 were enrolled for DFS and OS analyses. HCC was staged according to the criteria of the 7^th ^edition of the AJCC/UICC TNM staging system. All patients were followed regularly at 3-month intervals for clinical evaluation, laboratory data collection and imaging studies. The median follow-up period was 54.8 months. Of these 844 patients, 66.7% were positive for HBV infection, 38.5% were positive for HCV infection, and 57.7% had liver cirrhosis. Of those with liver cirrhosis, 93.4% were Child-Pugh class A.

**Table 1:** Clinicopathological characteristics of 879 patients with resectable hepatocellular carcinoma

	Number of patients (%) or median (25-75 percentile)
Age (years)	58 (47-66)

Male/female	707 (80.4)/172 (19.6)

Hepatitis B virus positive, hepatitis C virus positive	549 (66.7), 285 (38.5)

CTP status: A/B or C	821 (93.4)/58 (6.6)

ICG retention rate at 15 min (%)	9.7 (5.7-16.4)

Albumin (g/dL)	4.1 (3.8-4.4)

Bleeding (> 500 mL)	401 (45.9)

Mortality	35 (4.0)

AFP (ng/mL)	66.8 (9.7-770.0)

Tumor size (cm)	4.0 (2.5-7.0)

Rupture	53 (6.0)

Cirrhosis	507 (57.7)

Macro/microvascular invasion	188 (21.4)/104 (11.8)

Satellite lesions	229 (26.1)

Encapsulation	627 (71.3)

Resection margin positive	37 (4.2)

Grade	432 (49.1)/447 (50.9)

Well and moderate differentiated/poorly and undifferentiated	

CTP, Child-Pugh status; AFP, α-fetoprotein; Grade: Edmonson and Steiner; ICG, indocyanine green

**Figure 2:**
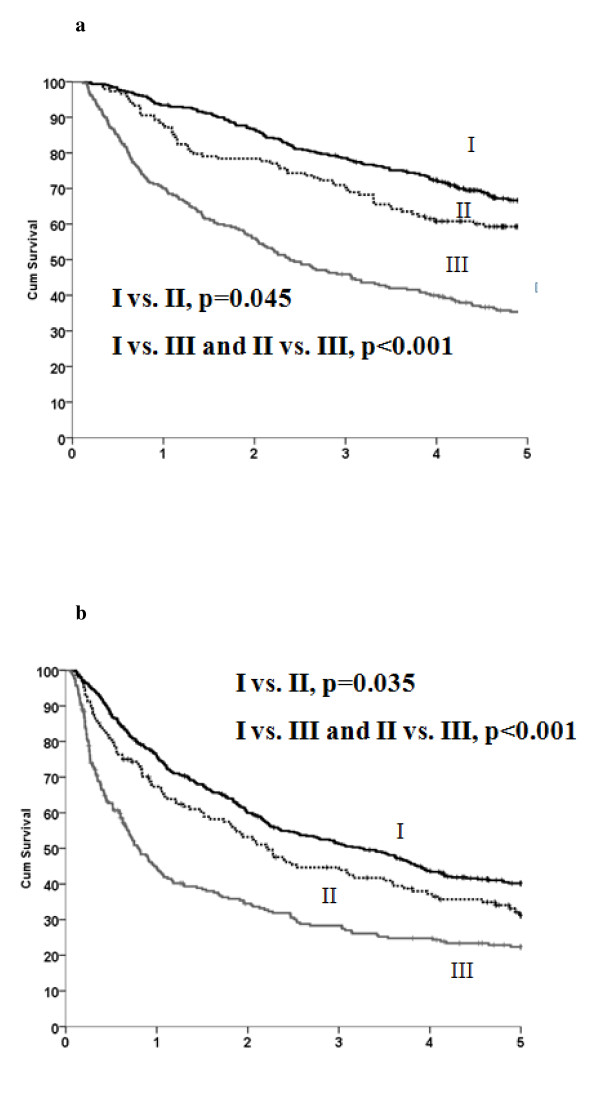
**Cumulative survival rates according to TNM-7 staging system of stages I, II and III**. **a**. Cumulative disease-free survival rates according to TNM-7 staging system of stages I, II and III. **b**. Cumulative overall survival rates according to TNM-7 staging system of stages I, II and III.

The 1-, 3-, 5-, 8-, and 10-year DFS rates in this series were 65.2%, 43.3%, 33.4%, 27.2%, and 25.8.0%, respectively, whereas the 1-, 3-, 5-, 8-, and 10-year OS rates were 85.3%, 67.2%, 54.7%, 40.0%, and 32.8%, respectively. After 5 years, statistically significant differences in survival were observed between patients with stages I, II, and III disease according to the TNM-7 (*p *< 0.05 for each group analysis; Figures 2a and 2b).

**Figure 3:**
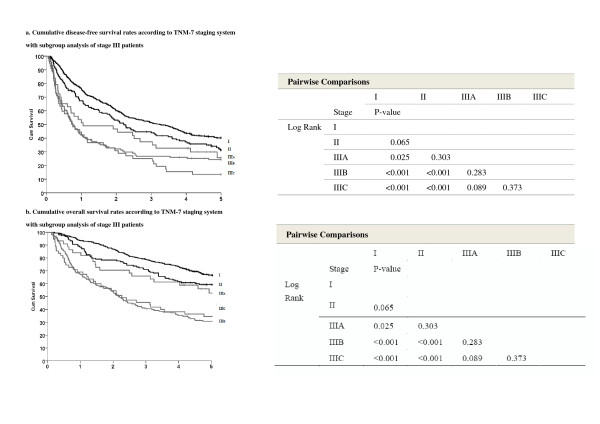
**Cumulative survival rates according to TNM-7 staging system with subgroup analysis of stage III patients**. **a**. Cumulative disease-free survival rates according to TNM-7 staging system with subgroup analysis of stage III patients. **b**. Cumulative overall survival rates according to TNM-7 staging system with subgroup analysis of stage III patients

Patients with stage III underwent further subgroup analysis. The 5-year OS and DFS were analyzed by pairwise comparison (Table 2, Figures 3a and 3b). Although some trends toward sub-classification of stage III HCC were apparent, differences between stages II and IIIA were not statistically significant (OS, *p *= 0.246; DFS, *p *= 0.105). Upon further stratification of stages IIIA, IIIB, and IIIC, differences remained statistically insignificant (Figure 3).

**Table 2:** Cox proportional hazard analysis of risk factors for 5-year overall and disease-free survival

	Overall survival			Disease-free survival		
	**HR**	**95% C.I.**	***p*-value**	**HR**	**95% C.I.**	***p*-value**

Vascular invasion	1.39	1.14-1.70	< 0.01	1.15	0.94-1.40	0.18

Satellite lesions	1.23	1.03-1.47	0.03	1.17	0.97-1.40	0.10

Rupture	1.53	0.98-2.40	0.06	1.21	0.76-1.91	043

Cirrhosis	0.90	0.66-1.21	0.47	0.96	0.70-1.30	0.80

Encapsulation	0.67	0.49-0.91	0.01	0.73	0.53-0.99	0.04

Grade (III, IV vs. I, II)	1.02	0.98-1.14	0.71	1.05	0.94-1.17	0.42

Albumin	0.97	0.64-1.47	0.88	0.75	0.49-1.14	0.17

AST (> 68 vs. ≤ 68 U/L)	1.84	1.30-2.61	< 0.01	1.65	1.14-2.40	0.01

Bil (> 1.3 ≤ vs. ( 1.3 mg/dL)	1.40	0.89-2.02	0.16	0.98	0.64-1.14	0.91

AFP (200 ng/mL)	1.44	1.05-1.98	0.02	1.28	0.94-1.75	0.123

Blood loss (> 0.5 L)	1.85	1.36-2.53	< 0.01	1.43	1.05-1.94	0.03

Margin involved	0.84	0.63-1.12	0.29	0.78	0.59-1.04	0.09

Grade: Edmonson and Steiner; AST, aspartate aminotransferase; Bil, total bilirubin; AFP, α-fetoprotein ICG, indocyanine green

### Cox proportional hazard models of stage III analysis

Subgroup analyses of 257 patients with stage III, 44 patients with stage IIIA, 158 patients with stage IIIB, and 55 patients with stage IIIC HCC were performed using the Cox proportional hazard model (Table 2). To identify additional important prognostic factors for stage III HCC, 12 clinicopathological factors including 6 pathological characteristics, 3 liver function tests, 2 surgical factors, and AFP values were analyzed. Lack of tumor encapsulation, AST values > 68 U/L, and blood loss > 500 mL were found to be independent significant prognostic factors affecting DFS. Moreover, lack of encapsulation, presence of vascular invasion, AST values > 68 U/L, blood loss > 500 mL, and AFP values > 200 ng/mL were found to be independent significant prognostic factors in the OS analysis. Stage III patients included those with tumor thrombus, satellite lesions, or rupture. Interestingly, these factors did not appear to be significant in the Cox proportional hazard model.

## Discussion

The AJCC/UICC TNM system is a widely used staging model for HCC patients. The most remarkable change in the 7^th ^edition is the dichotomization of stage IIIA by T3a and T3b (Figure 1). Findings of the present study, which intended to assess the validity of this new staging system for resectable HCC, revealed that this system was clearly capable of stratifying patients with stages I, II, and III in terms of 5-year survival rates. However, the TNM-7 failed to stratify stage III patients into stages IIIA, IIIB, and IIIC. The TNM-6 system was reported in 2006 to be superior to the TNM-5 system with respect to clinical relevance and prognostic value, but a surgical margin greater than 1 cm, ICG-R15 more than 10%, AST values > 90 U/L, and male gender were also found to be independent prognostic factors in multivariate analysis [22]. In the current evaluation of the TNM-7 staging system, stratification was not successful for stages III A-C by log-rank tests. Further analysis by the Cox proportional model disclosed that other factors, such as the lack of tumor encapsulation, AST values > 68 U/L, and blood loss > 500 mL, independently affected survival. These findings support the hypothesis that HCC patients usually present with other confounding factors that affect the long-term outcomes. A staging system should be capable of accounting for these factors and the most important drawback of the TNM-7 staging system is the lack of incorporation of host and surgical factors.

Staging systems are designed to predict prognosis and to define the most suitable treatment. Several staging classifications have been proposed, but currently no consensus exists regarding the best stratification for clinical practice [23-25]. Investigators utilizing the Akaike information criterion to compare 5 cancer staging systems among 1713 patients with early to advanced stages of HCC concluded that the CLIP staging system is the best long-term prognostic model and that its predictive accuracy is independent of treatment strategy [26]. In another investigation comparing 7 different staging systems for a cohort of HCC patients who underwent transarterial chemoembolization, the CLIP score was also found to provide the best prognostic stratification on the basis of the Akaike information criterion [27]. Almost all staging systems can stratify effectively in the context of a large scale patient population, but most staging systems have their own prediction inaccuracies. In a separate study comparing the BCLC, AJCC TNM-7, and Chinese staging systems, the Chinese and BCLC staging systems were found to be superior to the TNM-7 staging system in stratification and prognosis prediction. However, the subgroups of stage III patients were not well-stratified according to the TNM-7 classification [24]. The present study, which addresses the pros and cons of the TNM-7 system for resectable HCC, reveals that the accuracy of stratification is lost for the stage III population subgroup. Moreover, AFP values > 200 ng/mL, tumor encapsulation, and hepatitis (AST values > 68 U/L) were found to represent additional important factors affecting treatment outcome.

Liver function variables (ascites, bilirubin, alkaline phosphatase, and albumin concentrations) and host health status (male gender, performance state, and age) have also been reported to serve as major prognostic factors [28]. A unique characteristic of HCC is that the combination of viral infection, cirrhosis, and poor liver functional reserve also affects the outcome. Poor liver function reserve is an essential criterion for patient selection before resection. Consequently, patients with different liver function states but with the same TNM stage have different outcomes based on the probabilities of treatment. In the present study, cirrhosis was not an independent prognostic factor for stage III patients, although cirrhosis was associated with delayed recurrence of small HCC (data not shown). These findings are compatible with those of others [29].

Classification of stages I-II in the TNM-7 staging system did not change as compared to the TNM-6 staging system. In both systems, solitary lesions without vascular invasion and satellite lesions are classified as stage I and outcome is independent of tumor size. However, the association between tumor size and tumor aggressiveness is widely recognized [30]. Larger tumors (> 5 cm) are reported to be associated with greater likelihood of vascular invasion and higher histologic grading [31,32]. The biological behavior of tumors of different sizes and the outcome of patients with tumors of different sizes may not be uniform, and certainly the prognostic significance of tumor size requires further reevaluation.

In the present study, the 5-year DFS and OS rates were chosen as end points and the outcomes were compatible with those of other studies. Outcome differences between stages I-II and between stages II-III were statistically significant according to both the TNM-6 and the TNM-7 staging systems. However, the present study failed to discriminate outcome differences for stages IIIA, IIB, and IIIC. In this regard, it is of interest that staging systems currently under development are incorporating new biomarkers, such as lens culinaris agglutinin reactive AFP [33,34], des-c-carboxy prothrombin [35], glypican 3 [36], and osteopontin [37]. In the future, with regard to the goal of a more personalized medicine, the customization of scoring systems would ideally incorporate tumor pathological characteristics, host factors and, possibly, gene expression profiles. Improvements in the prognostic predictability of staging systems would redefine treatment strategies and such strategies may ultimately encompass gene and target therapies.

## Conclusions

In terms of 5-year survival rates, the 7^th ^edition of AJCC/UICC TNM system (TNM-7) effectively stratifies post-hepatectomy HCC patients into stages I, II, and III but is incapable of stratifying stage III patients into stages IIIA, IIIB, and IIIC. Lack of tumor encapsulation, AST values > 68 U/L, blood loss > 500 mL, and AFP values > 200 ng/mL are independent factors influencing the long-term survival of these patients.

## List of Abbreviations

AFP: α-fetoprotein; AJCC: American Joint Committee on Cancer; AST: aspartate aminotransferase; CT: computed tomography; DFS: disease-free survival; HCC: hepatocellular carcinoma; OS: overall survival; TNM: tumor-node-metastasis; UICC: International Union Against Cancer

## Competing interests

The authors declare that they have no competing interests.

## Authors' contributions

CHC and CFL: drafting the manuscript and data collection. THW, KMC, HSC, TJW: data collection. TCC: pathological review of surgical specimens. WCL, MFC: revising the manuscript. MCY: drafting and designing the manuscript, analysis and interpretation of data. All authors have seen and approved the final version to be published.
